# The Mediating Role of Empathy in the Relationship Between Mindfulness and Psychological Well-Being Among Special Education Teachers: Implications for Occupational Mental Health

**DOI:** 10.3390/healthcare14152396

**Published:** 2026-08-04

**Authors:** Hiam M. Alaoufi, Ahmed Mohamed Gadelrab Abouzaid, Salwa R. Saleh, Mahmoud Mohamed Eltantawy

**Affiliations:** 1 Department of Teaching and Learning, College of Education and Human Development, Princess Nourah bint Abdulrahman University, P.O. Box 84428, Riyadh 11671, Saudi Arabia; hmalaoufi@pnu.edu.sa; 2 Independent Researcher, Qena 83661, Egypt; drahmedab2020@gmail.com; 3 Department of Special Education, College of Education, Imam Mohammad Ibn Saud Islamic University (IMSIU), Riyadh 11432, Saudi Arabia; mmeltantawy@imamu.edu.sa

**Keywords:** mindfulness, empathy, psychological well-being, teacher, special education, mental health

## Abstract

**Background/Objectives:** Psychological well-being is an important indicator of occupational mental health among special education teachers, as it is closely associated with work engagement, professional performance, and attitudes toward students with disabilities. Despite its importance, limited research has examined factors associated with psychological well-being among special education teachers in Arab contexts. Therefore, this study examined the level of psychological well-being among special education teachers and investigated the relative contributions of mindfulness and empathy. In addition, the study tested the mediating role of empathy in the relationship between mindfulness and psychological well-being. **Methods:** A cross-sectional quantitative predictive correlational design was employed with a sample of 259 special education teachers in Riyadh, Saudi Arabia. Data were collected using an Arabic-adapted version of Ryff’s Psychological Well-Being Scale, the short form of the Five-Facet Mindfulness Questionnaire, and the Empathy Scale for Teachers. Path analysis was conducted to test a partial mediation model examining the mediating role of empathy in the relationship between mindfulness and psychological well-being. **Results:** The findings indicated high levels of psychological well-being among special education teachers. Mindfulness and empathy accounted for significant variance in psychological well-being, and path analysis showed that empathy partially mediated the relationship between mindfulness and psychological well-being. **Conclusions:** These findings highlight the potential importance of mindfulness and empathy in relation to the psychological well-being of special education teachers and suggest the value of giving greater attention to these dimensions in pre-service preparation and ongoing professional support programs. The study also contributes context-specific evidence from Saudi Arabia to the literature on teacher well-being in special education.

## 1. Introduction

Special education teachers face multiple professional demands that require continuous support and ongoing professional development [1]. They work in challenging educational settings, interact with heterogeneous groups of students with diverse needs, and are expected to help each student achieve individualized goals [2]. Within this demanding context, teachers’ occupational mental health has become an important issue, and recent research has highlighted the significance of teacher well-being and the factors associated with it [3,4,5]. Recent studies have increasingly emphasized teacher well-being as an occupational and relational construct linked to burnout, occupational stress, emotional exhaustion, work engagement, and professional sustainability [3,4,5]. Special education teachers have multiple responsibilities that often place them under considerable emotional and professional strain, which may be associated with adverse psychological outcomes and reduced professional functioning [6]. Burnout is one of the most frequently discussed indicators of such strain [3,7], and it has been associated with teachers’ intention to leave the profession [8]. In Saudi Arabia, burnout among special education teachers has been reported at 48.40% [9]. Previous research has also documented other mental health concerns in this population, including emotional exhaustion, anxiety, and depressive symptoms [10]. Taken together, these findings highlight the importance of examining not only negative occupational outcomes, but also positive indicators of adjustment, such as psychological well-being, and the personal resources that may be related to it [11,12]. In this regard, examining psychological well-being alongside potentially relevant personal resources, such as mindfulness and empathy, may contribute to a more comprehensive understanding of occupational mental health among special education teachers.

### 1.1. Psychological Well-Being

Psychological well-being is a fundamental aspect of human wellness and optimal functioning [13], and its promotion is closely linked to the satisfaction of basic psychological needs [14]. Psychological well-being is a mental condition that enables a person to reach their full potential, be creative and productive at work, and handle daily challenges [15]. Ryff [16] described psychological well-being as self-acceptance, positive relations with others, autonomy, environmental mastery, purpose in life, and personal growth.

There are many models that explain the dimensions of psychological well-being. Bakker and Oerlemans [17] developed a multi-level model of employee well-being consisting of three positive dimensions (work engagement, happiness at work, and job satisfaction) and two negative dimensions (workaholism and burnout). Positive Psychology has also introduced the PERMA model, a five-component framework of psychological well-being that includes positive emotion, engagement, relationships, meaning, and accomplishment [18]. This model is widely used as a contemporary framework for understanding psychological well-being. Psychological well-being has been conceptualized as a multidimensional construct that is difficult to define comprehensively [13]. Nevertheless, the literature does not fully agree on its core components and associated variables [19]. In some contexts, well-being is also conceptualized as the opposite of burnout and stress [19].

To address the complexity of defining psychological well-being, this study utilized Ryff’s Psychological Well-Being Scale. This instrument provides a clear operational framework by translating the multidimensional nature of well-being into six measurable dimensions, thereby supporting both conceptual clarity and empirical assessment.

Studies have emphasized the important role of psychological well-being and its association with several variables. A study by Greenier et al. [20] demonstrated the role of psychological well-being as a strong predictor of work engagement. Another study found that, among resilient traits, perseverance was significantly associated with psychological well-being and served as a mediator in this relationship [21]. Furthermore, Cenkseven-Onder and Sari [22] found that teachers’ subjective well-being was significantly associated with school quality of life and burnout-related factors.

Several studies have addressed the psychological well-being of special education teachers. For instance, Wu et al. [23] pointed to the mediating role of the Emotional Labor Perspective, while Bonner [24] found that competence and resilience were associated with psychological well-being, and underscored the broader relevance of resilience and self-efficacy to the well-being of special education teachers. Le et al. [25] found that life satisfaction, self-compassion, salary, and benefits all positively influence well-being levels.

### 1.2. Mindfulness

The concept of mindfulness has received considerable attention across schools of psychotherapy [26]. Mindfulness is defined as the ability to attend to and become aware of present moment experience without judgment or avoidance [27]. This approach may help create balance between thoughts and emotions, which is important for psychological adjustment [28]. Mindfulness has been widely used as evidence-based practice to support psychological well-being. Its benefits include reducing stress, increasing positive emotions, improving quality of life, and reducing depression and anxiety [29]. Recent teacher-focused evidence has further supported the relevance of mindfulness to teachers’ occupational mental health, particularly in relation to burnout, stress, anxiety, depression, and quality of life [29]. Mindfulness also contributes to preventing stress and burnout among service workers [30] and is one of the methods used to reduce anxiety and manage emotions [31]. It is also considered an aspect of social and emotional competence that may protect individuals from burnout and its negative mental, emotional, and physical consequences [32].

Numerous studies have shown that mindfulness training is associated with improved self-compassion and personal achievement, while also reducing levels of isolation, anxiety, fatigue, and emotional exhaustion [33,34]. Practicing mindfulness may enable individuals to develop self-compassion [35].

In previous research, participants in mindfulness-based stress reduction programs reported significant reductions in stress and anxiety and noticeable increases in positive affect and self-compassion [36]. These programs have also been found to have a positive impact on self-awareness and mindfulness strategies outside of sessions [37]. Recent evidence further indicates that mindfulness and meditation interventions can contribute positively to participants’ well-being [38], performance [39,40], and overall happiness [41]. In educational contexts, mindfulness practices may therefore be particularly relevant because teachers must manage emotional demands, sustain attention, and respond sensitively to students’ diverse needs.

In the context of teaching, findings indicate that mindfulness is positively associated with teachers’ quality of life and self-actualization, but negatively associated with anxiety, depression, and stress [29]. Mindfulness appears to play a protective role against burnout, although this effect may be less evident among highly stressed and ambitious teachers [32]. It has also been associated with greater resilience and may support teachers’ psychological health and performance [32]. In addition, training educators in mindfulness practices has been shown to improve their effectiveness and reduce burnout and stress [42]. Such practices may also provide self-help techniques that can be applied with special education students in classroom settings [43].

It has been found that psychological resilience may play a mediating role in the relationship between mindfulness and creative self-efficacy among special education teachers [44]. Recent research has documented a link between present moment awareness and the attention that characterizes mindfulness and the ability to relate positively to another individual’s emotions, knowledge, and perspectives [45].

In order to examine mindfulness among special education teachers, self-acceptance and perceived stress were used to investigate the relationship between mindfulness and burnout; the results showed that both had multiple significant serial mediation effects on this relationship [46]. Burnout is negatively related to mindfulness and perceived social support, and perceived social support is a partial mediator of the effect of mindfulness on special education teachers’ burnout [47]. Mindfulness among school teachers may also indirectly influence their attitudes toward inclusive education through empathy [48]. Higher levels of perceived empathy among teachers have also been associated with better mental health; lower stress, anxiety, and depression; and greater student engagement in educational activities [49]. Taken together, these findings suggest that mindfulness and self-compassion skills are important mechanisms for improving psychological well-being; however, further research is needed to confirm these findings [50].

### 1.3. Empathy

Empathy is a common interpersonal phenomenon and has attracted considerable research interest across various branches of psychology [51,52]. Accumulating evidence suggests that empathy is essential for developing and maintaining close interpersonal relationships and is an important motivational source for social behavior [53]. The concept of empathy has been of paramount importance in psychotherapy and has been identified as a key factor in therapeutic change [54].

In the literature, empathy has generally been conceptualized in two primary ways: affective empathy and cognitive empathy [55,56]. The first, derived from an affective perspective, refers to emotional responses to another person’s emotional experiences. It may also involve feeling something approximating, though not identical to, another person’s feelings, such as responding with concern when another person is sad [55]. The second conceptualization defines empathy from a cognitive perspective, referring to a person’s ability to understand others’ feelings by taking their perspective and interpreting their nonverbal cues [55,56].

Importantly, empathy includes both affective and cognitive components [57]. This allows for a more comprehensive understanding of empathy and its consequences: the affective component of empathy explains why we care about other people in need of our empathy and motivates us to respond sensitively, while the cognitive component explains what enables people to recognize and name others’ emotions [58]. Cognitive and affective empathy are intertwined and complement each other in explaining social behavior [59]. Essentially, empathy involves understanding what someone is thinking and feeling [58].

There are two important models of empathy: the Davis model [60], which defines empathy through the four dimensions of affective empathy, cognitive empathy, concern for others, and fantasy; and the Zaki model [61], which focuses on empathy as a dynamic social process involving the interaction between the person and the situation. Based on these models, two distinct lines of research based on empathy were developed.

The results of numerous studies have confirmed the importance of empathy in the educational process, as teachers’ empathy enhances their students’ learning [62] and enables them to recognize students’ feelings and emotions [55]. Emotional empathy motivates teachers to respond sensitively to their students’ emotional needs, providing comfort and encouragement [58,63]. Teachers who demonstrate empathy have been found to communicate more effectively with their students, build supportive relationships, and better motivate them [64]. Studies have also shown that certain dimensions of empathy (e.g., fantasy and personal distress) play a mediating role in the association between teachers’ attachment styles and their concerns about disability; moreover, a direct positive association between preoccupied attachment style, feelings, and concerns about disability has been reported [65]. Empathy among teachers also plays a positive role in reducing burnout, and empathic concern can negatively predict lower personal achievement [66]. High empathy is also associated with positive attitudes toward students with disabilities [67]. Empathy plays a mediating role between primary school teachers’ mindfulness traits and their attitudes toward inclusive education [48]. Affective empathy (e.g., positive involvement, suffering, empathy for others, and emotional contagion) positively influences physical and mental well-being [68].

Psychological well-being is a fundamental component of teachers’ professional quality of life, acting as a protective factor against stress and psychological disorders [69]. The literature indicates that psychological, cognitive, and social characteristics—primarily mindfulness and empathy—may contribute to improved well-being among teachers [23,24,25,39]. These variables are also important indicators for understanding human behavior and mental health [68].

### 1.4. Theoretical Integration of the Study

The present study is grounded in an integrated framework linking mindfulness, empathy, and psychological well-being among special education teachers. Central to this framework is Ryff’s multidimensional model of psychological well-being, which conceptualizes psychological well-being through six dimensions: self-acceptance, positive relations with others, autonomy, environmental mastery, purpose in life, and personal growth [13,16]. To explain the underlying mechanism, the study also draws on the self-regulation perspective of mindfulness, which views mindful awareness as a psychological resource that promotes non-judgmental attention to internal and external experiences [27,31].

Within this framework, mindfulness is assumed to influence psychological well-being both directly and indirectly through empathy. Empathy, informed by Davis’s multidimensional model of empathy [70], is understood as involving both cognitive and affective components, a distinction also emphasized by Baron-Cohen and Wheelwright [57]. More specifically, empathy includes Perspective Taking and Fantasy as cognitive dimensions, and Empathic Concern and Personal Distress as affective dimensions. From this perspective, mindfulness may be associated with greater Perspective Taking and Empathic Concern, as well as lower Personal Distress, which may, in turn, strengthen interpersonal functioning and support psychological well-being [45,61,68]. Accordingly, the study treats mindfulness, empathy, and psychological well-being as interrelated processes that together help explain the psychological well-being of special education teachers.

Although other personal resources, such as self-efficacy, emotion regulation, resilience, and self-compassion, are also relevant to teachers’ psychological well-being, empathy was selected as the mediator in the present study for both theoretical and contextual reasons. Theoretically, mindfulness involves non-judgmental present moment awareness and attentional regulation, which may enhance teachers’ capacity to notice, understand, and respond to students’ emotional and educational needs [27,31]. Previous research has also linked mindfulness-related present moment awareness to the ability to understand others’ emotions, knowledge, and perspectives [45]. Empathy is therefore positioned closer to the interpersonal pathway through which mindfulness may be translated into more adaptive teacher–student relationships and, consequently, better psychological well-being. This is particularly relevant for special education teachers, whose work requires continuous emotional attunement, perspective taking, and sensitive responses to students with diverse disabilities [55,57,70]. In addition, recent evidence suggests that empathy may mediate relationships involving teachers’ mindfulness and inclusive educational attitudes [48], and that empathy is positively associated with teachers’ mental health and well-being [49,68]. From this perspective, empathy provides a theoretically appropriate mechanism linking the intrapersonal resource of mindfulness with the relational and occupational dimensions of psychological well-being. In contrast, constructs such as self-efficacy and emotion regulation, although important, represent broader personal resources and were not the primary mechanism examined in the present study.

Despite the growing interest in teachers’ psychological well-being, important theoretical and practical gaps remain in the literature. Previous studies have emphasized the relevance of mindfulness and empathy to mental health and well-being among teachers and helping professionals [71]. In addition, theoretical perspectives such as Ryff’s model of psychological well-being and the PERMA framework suggest that attentional regulation, emotional regulation, and positive social orientations are important for psychological well-being [16,18]. However, limited research has examined the relative contribution of mindfulness and empathy to psychological well-being among special education teachers, particularly in Arab and Gulf contexts. Moreover, the mediating role of empathy in the relationship between mindfulness and psychological well-being remains insufficiently explored in this population. This represents a theoretical gap in understanding the mechanism through which mindfulness may be linked to psychological well-being among special education teachers.

This issue is particularly important in the Saudi context, where cultural and religious values may shape psychological concepts, interpersonal attitudes, and patterns of behavior [72,73,74]. Some studies in Arab societies have reported positive associations between religiosity and indicators of mental health, life satisfaction, and happiness [75]. These contextual influences may affect how mindfulness, empathy, and psychological well-being are understood, experienced, and expressed. However, religiosity and financial or socioeconomic factors were not directly measured in the present study; therefore, their potential influence is considered a contextual consideration rather than an empirically tested explanation. From a practical perspective, the limited availability of context-specific evidence makes it difficult to design effective pre-service and in-service programs that support the occupational mental health of special education teachers. Therefore, the present study contributes to the literature by examining the relative contribution of mindfulness and empathy to psychological well-being and by testing the mediating role of empathy among special education teachers in Saudi Arabia. Accordingly, the study addressed the following research questions:

(1) What is the level of psychological well-being among special education teachers in Saudi Arabia?
(2) To what extent do mindfulness and empathy explain variance in psychological well-being among special education teachers?
(3) Does empathy mediate the relationship between mindfulness and psychological well-being among special education teachers?

## 2. Materials and Methods

### 2.1. Study Design

The study adopted a cross-sectional, predictive correlational design, utilizing path analysis to examine the structural relationships among the study variables. This design was specified a priori to test a partial mediation model in which empathy was hypothesized to mediate the relationship between mindfulness and psychological well-being, while also allowing for a direct effect of mindfulness on psychological well-being. By employing this approach, the study goes beyond descriptive associations to evaluate the fit of the hypothesized mediation pathways, thereby providing a more rigorous statistical examination than simple correlation analysis.

### 2.2. Participants

Before collecting the data, we performed a statistical analysis using G*Power- (3.1.9.7) software to estimate the sample size required to analyze regression using two predictive variables: mindfulness and empathy. We assumed the following values to calculate regression (effect size f^2^ = 0.15, α error probability = 0.05, and power (1 − β) = (0.95)). The analysis showed that we needed at least 107 participants. The path analysis was based on Kline’s [76] assertion that models based on the maximum likelihood estimate need at least 200 participants to ensure the stability of the parameters and the impartiality of the results.

The study sample consisted of 259 male and female special education teachers, selected using a convenience sampling method from a population of 3905 special education teachers in Riyadh, Saudi Arabia, representing 6.63% of the target population according to the 2023 statistics of the Saudi Ministry of Education. Participants were recruited from schools offering special education programs at the primary and middle school levels. These schools included approximately 420 teachers, of whom 259 completed the electronic questionnaire, yielding a response rate of 61.7%. The final sample exceeded the minimum required sample size, supporting the adequacy of the statistical analyses. Although high school special education teachers were initially included in the data collection process, they were excluded from the final analysis because only six responses were obtained, which was insufficient for meaningful representation or reliable subgroup analysis. Accordingly, the final analysis was restricted to primary and middle school teachers to improve sample consistency and ensure more robust statistical interpretation. No missing data were identified, as all questionnaire items were configured as mandatory in the electronic survey. Table 1 shows the demographic characteristics of the participants.

### 2.3. Tools

To achieve the objectives of the study, a set of measurement instruments was employed. These instruments were selected for their relevance to the study variables, their established psychometric properties in previous research, and their suitability for assessing empathy, mindfulness, and psychological well-being in the context of the present study. Moreover, their Arabic versions have been validated in similar cultural settings, and their concise format helps minimize survey fatigue among special education teachers working in demanding educational environments.

#### 2.3.1. Ryff’s Psychological Well-Being Scale

The short version of the Ryff scale consists of 18 items, with three items representing each of the six dimensions. The subscales of the short version have shown correlations ranging from 0.70 to 0.89 with their corresponding subscales in the original 120-item version. The scale is a self-report measure of psychological well-being, encompassing six dimensions: autonomy, environmental mastery, self-acceptance, personal growth, positive relationships with others, and purpose in life [13]. Responses are provided on a five-point Likert scale ranging from 1 to 5.

##### Translation, Cultural Adaptation, and Face Validity

In the current study, the scale was translated into Arabic by four specialists in psychology. The translated versions were reviewed and reconciled into a single preliminary Arabic version. This version was then back-translated into English by an independent translator to ensure linguistic accuracy. The back-translated version was compared with the original English scale, and any discrepancies in meaning were identified and revised accordingly.

Following the translation process, the Arabic version was presented to a group of 12 teachers from the target population to assess the clarity of the wording, the comprehensibility of the items, and the appropriateness of the language used. Based on their feedback, minor modifications were made to enhance clarity and accuracy. After finalizing the Arabic version, the psychometric properties of the scale, including validity and reliability, were examined.

##### Confirmatory Factor Analysis (CFA) and Reliability

The construct validity and reliability of the Psychological Well-Being Scale were examined using CFA and internal consistency measures, with all analyses conducted on the entire study sample (N = 259). The CFA results indicated that the proposed measurement model demonstrated a good fit to the data. Specifically, the fit indices were within the recommended thresholds, with the Comparative Fit Index (CFI = 0.959) and Tucker–Lewis Index (TLI = 0.947) exceeding the acceptable cutoff, and the Root Mean Square Error of Approximation (RMSEA = 0.061) indicating an adequate fit, consistent with the criteria suggested by Hu and Bentler [77]. Furthermore, all standardized factor loadings were statistically significant and ranged from 0.661 to 0.840, reflecting strong and adequate associations between the observed items and their corresponding latent constructs [78].

In addition, convergent validity was supported by the Average Variance Extracted (AVE), with values ranging from 0.61 to 0.79, all exceeding the recommended minimum threshold of 0.50 [79]. Composite Reliability (CR) values ranged from 0.77 to 0.92, indicating satisfactory to high construct reliability. Reliability was further confirmed using Cronbach’s alpha, with coefficients ranging from 0.76 to 0.92 across subscales and reaching 0.908 for the overall scale, exceeding the acceptable threshold of 0.70 [80].

#### 2.3.2. Five-Facet Mindfulness Questionnaire (FFMQ): Short Form

The FFMQ was developed by Baer et al. [81] and is a 39-item self-report measure consisting of five factors: observe, describe, act with awareness, non-judge, and non-react. The current study used the short form of the questionnaire, which consists of 20 items and whose psychometric properties were calculated by Tran et al. [82]. It measures the same five factors and was standardized on two independent samples (first sample: 640 participants aged 18–76 years; second sample: 333 students aged 18–41 years). Exploratory and confirmatory factor analyses revealed two measuring factors, and the shortened scale achieved adequate correlation with the original version and with other constructs. The internal consistencies (Cronbach’s alpha) in the shortened form were 0.68 (observation), 0.79 (description), 0.78 (action awareness), 0.78 (non-judgment), and 0.48 (non-reaction) in the student sample. Responses are provided on a five-point Likert scale ranging from 1 to 5.

##### Translation, Cultural Adaptation, and Face Validity

The questionnaire was translated into Arabic by four specialists in psychology after obtaining permission to adapt it for use in the Arab context. The translated versions were then reviewed and reconciled into a single preliminary Arabic version. This version was subsequently back-translated into English by an independent translator to ensure linguistic accuracy. The back-translated version was compared with the original English questionnaire, and any discrepancies in meaning were identified and revised accordingly.

Following the translation process, the Arabic version was presented to a group of 13 teachers from the target population to assess the clarity of the wording, the comprehensibility of the items, and the appropriateness of the language used. Based on their feedback, minor modifications were made to improve clarity and accuracy. After finalizing the Arabic version, the psychometric properties of the questionnaire, including validity and reliability, were examined.

##### CFA and Reliability

The measurement properties of the scale were examined through CFA and internal consistency statistics using the full study sample (N = 259). The analysis showed that the proposed model fit the data adequately, as indicated by the CFI (0.931), TLI (0.918), and RMSEA (0.070). All standardized factor loadings were statistically significant and ranged from 0.706 to 0.857, suggesting that the items were suitably aligned with their intended latent factors. Convergent validity was further supported by AVE values ranging from 0.52 to 0.66, all above the 0.50 criterion. CR values ranged from 0.74 to 0.89, indicating satisfactory to strong reliability. Cronbach’s alpha coefficients ranged from 0.73 to 0.89 across the subscales and reached 0.91 for the total scale, reflecting acceptable internal consistency.

#### 2.3.3. Empathy Scale for Teachers (EST)

Wang et al. [53] developed the Empathy Scale for Teachers, which comprises 19 items across three dimensions: cognitive empathy, positive affective empathy, and negative affective empathy. Responses are provided on a five-point Likert scale ranging from 1 (completely disagree) to 5 (completely agree). The scale’s psychometric properties were supported through exploratory and confirmatory factor analyses, with the instrument demonstrating satisfactory validity and reliability [53].

##### Translation, Cultural Adaptation, and Face Validity

In the current study, the scale was translated into Arabic by four specialists in psychology. The translated versions were reviewed and reconciled into a single preliminary Arabic version. This version was then back-translated into English by an independent translator to ensure linguistic accuracy. The back-translated version was compared with the original English scale, and any discrepancies in meaning were identified and revised accordingly.

Following the translation process, the Arabic version was presented to a group of 17 teachers from the target population to assess the clarity of the wording, the comprehensibility of the items, and the appropriateness of the language used. Based on their feedback, minor modifications were made to improve clarity and accuracy.

##### CFA and Reliability

The psychometric adequacy of the scale was also supported by CFA and internal consistency indicators using the full study sample (N = 259). The results confirmed an acceptable model fit, with CFI values of 0.961, TLI = 0.947, and RMSEA = 0.080. In addition, the standardized factor loadings were all significant and ranged from 0.680 to 0.872, indicating that the observed items loaded well on their corresponding latent constructs. Evidence of convergent validity was demonstrated by AVE values ranging from 0.62 to 0.82, which exceeded the recommended minimum of 0.50. CR values ranged from 0.88 to 0.98, showing high construct reliability. Internal consistency was also strong, as Cronbach’s alpha coefficients ranged from 0.88 to 0.98 across the subscales and reached 0.973 for the overall scale.

### 2.4. Data Collection

After obtaining approval from the Institutional Review Board (IRB) of Princess Nourah bint Abdulrahman University, Riyadh, Kingdom of Saudi Arabia (IRB Log Number: 25-0116; IRB Registration Number with KACST: HAP-01-R-059; Date of Approval: 2 March 2025) and authorization from the Saudi Ministry of Education to use the three study instruments with teachers, the instruments were consolidated into a single electronic form via Google Forms. The Ministry of Education distributed the form link to all schools in Riyadh through official emails, and the school directors further shared the link with their teaching staff via their schools’ WhatsApp groups, ensuring that all teachers had an equal opportunity to respond to the questionnaires. The teachers were asked to consent to the use of their responses to achieve the study’s objectives. They were also made aware of the purpose of the study and that they could withdraw at any time without justification. To ensure objectivity and reduce participant bias, the submission procedure was standardized in terms of digitally provided instructions, while ensuring confidentiality of identity and data and avoiding direct interaction while completing tasks. All participants worked in public schools during the 2024–2025 school year.

### 2.5. Statistical Methods

During the data analysis phase, SPSS version 27 and AMOS 24 were used. The reliability of the study instruments was evaluated using Cronbach’s alpha coefficients. The normality of the data was examined using the Kolmogorov–Smirnov and Shapiro–Wilk tests, where non-significant results (*p* > 0.05) were interpreted as indicating no significant departure from normality [83,84]. A one-sample *t*-test was conducted to compare the observed mean scores of psychological well-being with the hypothetical mean derived from the midpoint of the scale. Multiple regression analysis was performed to examine the extent to which mindfulness and empathy accounted for variance in psychological well-being. Regression diagnostics included confidence intervals, tolerance values, variance inflation factor (VIF), and the Durbin–Watson statistic to assess multicollinearity and the independence of residuals [85,86]. In addition, path analysis was conducted to test a mediation model in which empathy was specified as a mediator in the association between mindfulness and psychological well-being.

Across all research questions, the total scale scores were used as the primary units of analysis. The total scores for mindfulness, empathy, and psychological well-being were calculated for each participant and subsequently used in the statistical analyses, including descriptive statistics, Pearson correlation analysis, and mediation testing. This approach was adopted to examine the overall relationships among the main study constructs.

## 3. Results

To verify the validity of the first question, “What is the level of psychological well-being among special education teachers in Saudi Arabia?”, the means and standard deviations of the Psychological Well-Being Scale and its sub-dimensions were calculated. A one-sample *t*-test was used to determine whether the observed mean differed significantly from the hypothetical mean. Table 2 shows the results for the first question.

To address the second research question, “To what extent do mindfulness and empathy account for variance in psychological well-being among special education teachers?”, a multiple linear regression analysis using the Enter method was conducted [86]. Mindfulness and empathy were entered as the independent variables, and psychological well-being was specified as the dependent variable. The results are presented in Table 3, Table 4 and Table 5.

Table 3 presents the descriptive statistics and Pearson correlation coefficients for the study variables. Psychological well-being was positively correlated with mindfulness (r = 0.570, *p* < 0.01) and empathy (r = 0.664, *p* < 0.01), while mindfulness was also positively correlated with empathy (r = 0.545, *p* < 0.01). These correlations indicated that higher scores on mindfulness and empathy were associated with higher psychological well-being.

As shown in Table 4, the overall regression model was statistically significant (F = 129.108, *p* < 0.001), and the predictor variables together accounted for 50.2% of the variance in psychological well-being (R^2^ = 0.502; Adjusted R^2^ = 0.498). The Durbin–Watson value of 1.980 suggested that autocorrelation of residuals was not a concern.

Table 5 shows that both mindfulness and empathy were significantly associated with psychological well-being when entered simultaneously into the model. Empathy had the stronger standardized association (β = 0.503, *p* < 0.001), whereas mindfulness also showed a statistically significant association with psychological well-being (β = 0.296, *p* < 0.001). This indicates that, after accounting for the shared variance between the predictors, higher empathy and higher mindfulness scores were each associated with higher levels of psychological well-being.

To answer the third research question, “Does empathy mediate the relationship between mindfulness and psychological well-being among special education teachers?”, a path analysis was conducted using AMOS 24. The analysis was used to estimate the direct association between mindfulness and psychological well-being, as well as the indirect association between them through empathy. Because the proposed mediation model was just-identified, the evaluation did not rely on global goodness-of-fit indices. Instead, interpretation focused on the magnitude and statistical significance of the structural path coefficients, including the direct, indirect, and total effects. The results are presented in Table 6.

The path analysis results presented in Table 6 show that all direct paths in the model were statistically significant. Mindfulness was positively associated with empathy (β = 0.545, *p* < 0.001). In addition, both empathy and mindfulness were positively associated with psychological well-being, with standardized coefficients of β = 0.503 and β = 0.296, respectively. The association between empathy and psychological well-being was stronger than the direct association between mindfulness and psychological well-being.

The indirect association between mindfulness and psychological well-being through empathy was statistically significant (β = 0.274), as the 95% bias-corrected bootstrap confidence interval did not include zero. The total association between mindfulness and psychological well-being was β = 0.570. Because the direct association between mindfulness and psychological well-being remained statistically significant after including empathy in the model, the findings support partial mediation. In other words, empathy statistically explained part of the association between mindfulness and psychological well-being among special education teachers.

**Figure 1:**
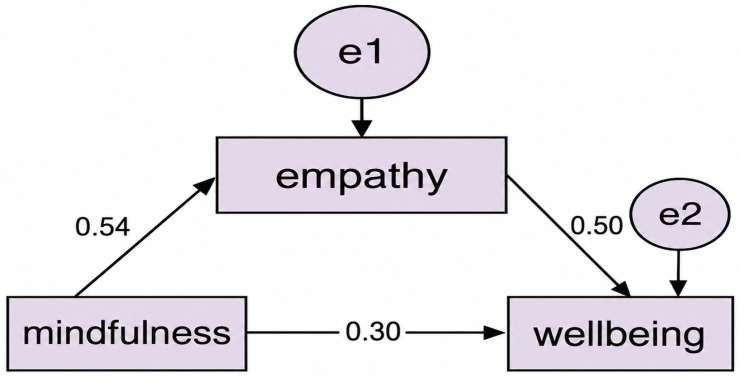
Structural model showing the relationship between mindfulness, empathy, and psychological well-being; e1c and e2 represent error terms.

To provide a complete visual representation of these relationships, Figure 1 illustrates the final structural model, including the standardized path coefficients that indicate the strength and direction of the direct and indirect associations among the study variables.

## 4. Discussion

The results for the first question indicate a high level of psychological well-being among special education teachers. These findings are consistent with those of the study by Al-Shaikhi and Khalifa [87], which indicated a high level of psychological well-being among female special education teachers in Jeddah, Saudi Arabia. Moreover, the results reflect those of Othman and Ahmed [88], who found a high level of psychological well-being among male and female teachers working in special needs centers in Zliten, Libya. There are also similarities with the findings from Akrama et al. [89], which indicated a high level of psychological well-being among special education teachers in Pakistan.

These findings may be interpreted in light of the multidimensional nature of psychological well-being, which is influenced by personal, relational, and occupational factors [19]. In the present study, the high level of psychological well-being among special education teachers should be understood primarily in relation to the study variables and the descriptive findings, rather than as evidence of unmeasured contextual causes. Previous research has suggested that school quality of life may be associated with teachers’ well-being and may support personal, professional, and social growth [22]. In the Saudi context, broader contextual factors, such as institutional support, cultural values, religiosity, and financial or socioeconomic conditions, may also be relevant to teachers’ well-being. Previous studies have also reported associations between psychological well-being and socioeconomic, religious, and spiritual factors [90,91,92]. However, these variables were not directly measured in the present study. Therefore, they are presented only as possible contextual considerations and should not be interpreted as empirically tested explanations for the high level of psychological well-being observed in this sample. Future studies should include direct measures of religiosity, financial satisfaction, socioeconomic status, and perceived institutional support to examine their potential contribution to psychological well-being among special education teachers.

The results of the second question showed that empathy was positively correlated with psychological well-being among special education teachers. These findings are consistent with those reported by Vinayak and Judge [93], who found that empathy was positively associated with psychological well-being and served as a significant indicator. The findings are also consistent with those of several previous studies [94,95,96,97] that likewise reported a positive association between empathy and psychological well-being. The result for this question can be interpreted in light of the fact that empathy correlates with an individual’s sense of others and ability to view from their perspective, which develops positive and constructive social relationships between themselves and others. It also increases self-esteem and self-confidence, which enhances the ability to set goals and achieve them. This positively impacts a person’s feelings of happiness and satisfaction, which, in turn, leads to a sense of psychological well-being.

The results also indicate that mindfulness contributes to predicting psychological well-being among special education teachers. One possible explanation is that mindfulness helps teachers to see their work environment more positively, reduces burnout, and promotes a more positive approach to professional challenges. It is also associated with improvements in mental and physical health, including reduced anxiety, depression, and stress, as well as better emotional regulation. These benefits enhance teachers’ overall satisfaction and happiness, boost productivity, support personal growth and independence, and foster positive relationships with colleagues—all of which contribute to their psychological well-being; these positive outcomes are supported by several other studies [28,29,30,31,35,39].

The results of the third research question indicated that empathy serves as a partial mediator in the relationship between mindfulness and psychological well-being among special education teachers. This finding is generally consistent with previous research suggesting links among mindfulness, empathy, and positive psychological and educational outcomes. For example, Jennings [71] associated teachers’ mindfulness with greater emotional support, perspective-taking, and sensitivity in classroom interactions. More specifically, several studies suggest that empathy may function as a mechanism through which mindfulness relates to important outcomes. Huang [48] found that empathy mediated the relationship between primary school teachers’ trait mindfulness and their attitudes toward inclusive education. Similarly, Wang et al. [98] reported that empathy, together with emotional intelligence, played a chain-mediating role in the relationship between preschool teachers’ trait mindfulness and teacher–child relationship quality. In addition, Pérez-Fuentes et al. [99] showed that cognitive empathy mediated the association between mindfulness and engagement among healthcare workers, providing broader support for the mediating role of empathy across helping professions. Braun et al. [100] also provided supporting evidence, showing that teachers’ mindfulness skills were associated with empathy development and improved well-being.

This pattern may suggest that teachers with higher mindfulness are more likely to respond empathically to students’ needs, which may, in turn, support their psychological well-being. This interpretation may be particularly relevant for special education teachers, whose work requires sustained emotional engagement, sensitivity to diverse student needs, and frequent interpersonal interaction in demanding educational settings. Psychological well-being, as conceptualized by Ryff and Keyes [13], includes self-acceptance, positive relations with others, autonomy, environmental mastery, purpose in life, and personal growth. In this sense, the present finding is also compatible with broader theoretical perspectives that emphasize positive relationships as a central component of well-being [18].

Although the present findings are broadly consistent with previous studies, they should be interpreted with caution. Differences across teacher populations, occupational demands, cultural contexts, and measurement approaches may partly explain convergence or divergence across studies. In addition, because the current findings are based on statistical associations, they should be understood as indicative rather than causal.

The current study adds value to the psychological literature by providing evidence from an underrepresented Arab and Saudi educational context. In this context, cultural and religious values may shape how empathy and psychological well-being are understood, experienced, and expressed, although these factors were not directly measured in the present study. The study also specifically involved special education teachers, thus differing from previous studies that mostly focused on other categories of teachers. In addition, the study results provide context-specific recommendations for professional development and teacher support, while identifying the variables that showed the strongest statistical association with teachers’ psychological well-being.

Several limitations should be considered when interpreting and generalizing the findings of the present study. First, the study focused exclusively on special education teachers and did not include general education teachers, which limits the generalizability of the findings to broader teaching populations. Second, data were collected using self-report questionnaires, which may introduce participant response bias. Relatedly, because all variables were measured using self-report instruments collected from the same participants at a single point in time, the findings may be subject to common method bias, which could inflate the observed associations among mindfulness, empathy, and psychological well-being. Third, the sample size was relatively small compared with the overall study population, and the use of convenience sampling further limits the generalizability of the results. Fourth, although the translated measures were reviewed and psychometrically examined, the use of instruments originally developed in Western cultural contexts may have resulted in some loss of cultural or linguistic nuance. These measures may not fully capture the cultural and social specificities of special education teachers in the Saudi context. Finally, some potentially relevant contextual variables, such as socioeconomic status, financial satisfaction, religiosity, and perceived institutional support, were not directly measured in the present study. Future research should address these limitations by using larger and more representative samples, including teachers from different educational settings, employing longitudinal or multi-source designs, and incorporating direct measures of relevant contextual variables.

## 5. Conclusions and Recommendations

The study findings indicated relatively high levels of psychological well-being among special education teachers. The results also showed that mindfulness and empathy were significantly associated with psychological well-being, while empathy was found to function as a partial mediator in the relationship between mindfulness and psychological well-being. These findings highlight the potential relevance of mindfulness and empathy among special education teachers as resources that may support psychological well-being, encourage acceptance and constructive interaction with students with disabilities, and contribute to professional effectiveness.

In addition, the study translated three instruments into Arabic and confirmed their reliability, which supports their appropriateness for use in the Saudi context. The findings may therefore provide a useful basis for subsequent research in this area. Future studies are encouraged to examine other variables related to psychological well-being among special education teachers using alternative methodological approaches, including qualitative and mixed-methods designs. It would also be valuable to include teacher groups that were not represented in the current study, such as kindergarten and secondary school special education teachers. Moreover, future researchers are encouraged to employ sampling strategies other than convenience sampling, particularly stratified random sampling, in order to obtain a more representative sample from different regions of Saudi Arabia.

From an applied perspective, the findings should be viewed as preliminary and hypothesis-generating. The study suggests that the Ministry of Education may consider developing training programs aimed at supporting mindfulness and empathy among special education teachers. In addition, professional development and teacher preparation processes may benefit from giving greater attention to these dimensions. Finally, further attention to the organizational environment of special education programs may also be valuable and should be examined in future research.

## Figures and Tables

**Table 1:** Teachers’ demographic characteristics (N = 259).

Variables	Category	Frequency (F)	Percentage (%)
Gender	Male	95	36.7
	Female	164	63.3
School Stage	Primary School	177	68.3
	Middle School	82	31.7
Disability Category	Intellectual Disability	93	35.9
	Autism Spectrum Disorder	78	30.1
	Learning Difficulties	48	18.5
	Hearing Impaired	30	11.6
	Visually Impaired	10	3.9
Age	20–30	27	10.4
	31–40	102	39.4
	41–50	94	36.3
	50+	36	13.8
Years of Experience	Less than 5	20	7.7
	5–10	78	30.1
	11–15	90	34.7
	More than 15	71	27.4
Qualification	Bachelor	234	90.3
	Postgraduate studies	25	9.7

**Table 2:** One-sample *t*-test for differences between the arithmetic mean and the hypothetical mean on the psychological well-being scale.

Variable	N	Mean	Std. Deviation	Min	Max	Hypothetical Mean	t	*p*
Positive Relations	259	11.0425	2.63587	3.00	15.00	9	12.470	*p* < 0.001
Environmental Mastery	259	12.0965	2.20340	5.00	15.00	9	22.617	*p* < 0.001
Self-Acceptance	259	11.5135	2.72990	5.00	15.00	9	14.818	*p* < 0.001
Autonomy	259	12.2317	2.26543	6.00	15.00	9	22.958	*p* < 0.001
Personal Growth	259	11.5097	2.51717	4.00	15.00	9	16.045	*p* < 0.001
Purpose in Life	259	10.7876	2.39454	4.00	15.00	9	12.015	*p* < 0.001
Total Well-Being	259	69.181	10.129	37.00	85.00	54	24.120	*p* < 0.001

Note: The hypothetical mean was calculated based on the midpoint of the scale. Each subscale consisted of three items rated on a five-point Likert scale, yielding a hypothetical mean of 9 for each dimension. The total psychological well-being score consisted of six dimensions, resulting in a hypothetical mean of 54. A one-sample *t*-test was conducted to compare the observed means with the hypothetical means. The results indicate that the arithmetic means for the total score and all subdimensions were significantly higher than the hypothetical means (*p* < 0.001), reflecting a high level of psychological well-being among special education teachers.

**Table 3:** Descriptive statistics and Pearson correlation matrix for study variables.

Variable	M	SD	Range	1	2	3
Psychological Well-Being	69.181	10.129	48.00	(1)		
Mindfulness	72.660	12.374	60.00	0.570 **	(1)	
Empathy	77.104	11.947	68.00	0.664 **	0.545 **	(1)

Note: ** *p* < 0.01.

**Table 4:** Summary of multiple linear regression model explaining the variance in psychological well-being (N = 259).

Model	Predictors	R	R^2^	Adjusted R^2^	Std. Error	F	*p*	Durbin–Watson
1	Mindfulness, Empathy	0.709 a	0.502	0.498	7.175	129.108	*p* < 0.001	1.980

a. Predictors: (Constant), empathy, mindfulness.

**Table 5:** Multiple linear regression coefficients for psychological well-being.

Model	Predictor	B	SE	β	t	*p*	95% CI Lower	95% CI Upper	Tolerance	VIF
1	(Constant)	18.73	3.17	—	5.90	*p* < 0.001	12.48	24.96	—	—
1	Mindfulness	0.242	0.043	0.296	5.62	*p* < 0.001	0.157	0.327	0.703	1.42
1	Empathy	0.426	0.045	0.503	9.56	*p* < 0.001	0.338	0.514	0.703	1.42

**Table 6:** Direct, indirect, and total effects of mindfulness on psychological well-being through empathy.

Path	Effect	B	SE	β	CR	*p*	95% CI for β Lower	95% CI for β Upper
Mindfulness → Empathy	Direct	0.526	0.050	0.545	10.44	<0.001	0.446	0.632
Empathy → Well-Being	Direct	0.426	0.044	0.503	9.59	<0.001	0.409	0.592
Mindfulness → Well-Being	Direct	0.242	0.043	0.296	5.64	<0.001	0.213	0.376
Mindfulness → Empathy → Well-Being	Indirect	0.224	0.044	0.274	5.09	<0.001	0.196	0.354
Mindfulness → Well-Being	Total Effect	0.466	0.054	0.570	8.62	<0.001	0.468	0.656

Note: Unstandardized coefficients (B), standard errors (SE), standardized coefficients (β), and bootstrap confidence intervals are reported. The 95% bias-corrected confidence intervals were generated using 5000 bootstrap samples and refer to the standardized estimates (β).

## Data Availability

The data presented in this study are available on request from the corresponding author. The data are not publicly available due to privacy and ethical restrictions.
